# Supplementary material for: The Mediterranean Diet Scale (MDS): Translation and validation of the Arabic version
Source: PLoS One. 2023 Aug 24;18(8):e0287497. doi: 10.1371/journal.pone.0287497 (PMC10449107; doi:10.1371/journal.pone.0287497)
Supplement: S1 File — (DOCX) [file pone.0287497.s001.docx]

**The Mediterranean Diet Scale (MDS):**

**translation and validation of the Arabic version**

**Authors:** Raghdah Aljehani,^1^ Ghaidaa Aljehani,^1^ Hanaa Alharazi,^2^ Paula M Horta,^3^ Camila Kümmel Duarte,^3^ Gabriela Lima de Melo Ghisi.^4,5*^

**Affiliations:**

1. Rehabilitation Department, King Abdullah Medical City, Makkah, Saudi Arabia.

2. Cardiology Department, King Abdullah Medical City, Makkah, Saudi Arabia.

3. Department of Nutrition, Federal University of Minas Gerais, Belo Horizonte, Brazil.

4. Department of Physical Therapy, University of Toronto, Toronto, Canada.

5. KITE, Toronto Rehabilitation Institute, University Health Network, Toronto, Canada.

***Corresponding author:**

Department of Physical Therapy, University of Toronto, Toronto, Canada.

160-500 University Ave, Toronto, Canada M5G 1V7

Tel: (416) 597-3422 x. 5261 Fax: (416) 425-0301

email: [gabriela.meloghisi@uhn.ca](mailto:gabriela.meloghisi@uhn.ca)

**Abstract**

Objectives: The self-administered version of the Mediterranean Diet Scale (MDS) has been developed to test the inherent characteristics of this dietary pattern in a quick and simple way, due to the need of this assessment in the clinical and research setting. This study aimed to translate and psychometrically validate the self-administered MDS in Arabic (CRBS-A).

Methods: The original (English) version was originally translated to Arabic, followed by back-translation. Next, 10 healthcare providers, followed by 10 cardiovascular disease (CVD) patients rated the face and content validity (CV) of materials, providing input to improve cross-cultural applicability. Then, 200 patients from Saudi Arabia completed the questionnaire, of which factor structure, internal consistency, criterion and construct validity were assessed.

Results: Content and face validity was supported based on experts and patients’ reviews (ranges: CV scores 0.9-1.0/1.0 and clarity 3.5 to 4.5/5). Minor edits were made. Subsequent factor analysis revealed 4 factors consistent with the original version of the instrument, all internally consistent. Total CRBS-A α was 0.74. Criterion validity was confirmed by the significantly higher scores in patients who participated in CR. Construct validity was also established by significant associations between MDS scores and monthly family income, having the diagnosis of acute coronary syndrome or with a history of valve repair or replacement, being obese or having dyslipidemia.

Conclusions: Overall, these results confirm the validity and reliability of the MDS in Arabic-speaking patients.

**Keywords:** Questionnaires and Surveys; Psychometrics; Diet, Mediterranean; Cardiac Rehabilitation; Coronary Artery Disease/Prevention and Control; Diet Surveys.

**Introduction**

Cardiovascular diseases (CVDs) are the most notable causes of disease burden across many nations [1], including Arabic-speaking countries [2,3]. In addition to health impacts, CVDs also carry a substantial economic burden on patients, their families, and societies due to medical costs, diminished productivity from disability and premature death [4-6]. Cardiovascular associations - regionally and internationally – recommend secondary prevention interventions to address this burden, which rely on successful health behaviour change [7-10]. These recommendations are based on findings linking CVDs to modifiable risk factors, particularly unhealthy behaviours such as poor-quality diet [10-12].

Cardiac rehabilitation (CR) is a comprehensive outpatient chronic disease management program designed to facilitate heart-health behaviours [9]. CR typically includes core components that address CVD risk factors, including exercise training, patient education, psychosocial management risk factor modification and nutritional counseling [13]. Research has shown that when all these multifactorial components are delivered, CR participation is associated with reductions of mortality up to 25%, as well as beneficial effects on morbidity, symptoms, exercise tolerance and capacity, lipid and blood pressure levels, blood pressure, psychosocial functioning and behaviour change [14-16]. In order to tailor adequate CR strategies and sustainable recommendations, it is crucial to fully understand patients’ habits that determine cardiovascular health [17]. One of those is an adequate nutrition [18].

Over the years, various dietary patterns have been studied with the goal to identify the one that is mostly effective on heart health. The Mediterranean Dietary Pattern (MDP) has been greatly studied, with evidence showing its effects on the prevention and treatment of CVDs, as well as on the reduction of CVD mortality (MDP) [19-21]. While the MDP is widely recommended to reduce CVD risk, there is not robust evidence for adherence in CR after the many changes that happened in programs due to the COVID-19 pandemic [22,23].

In this context, questionnaires are considered useful means to collect important information that can support choices related to the care of patients [24]; however, not always their application can be implemented into clinical practices. For example, full-length food frequency questionnaires (FFQ) or food records used in dietary assessments have limitations related to accuracy and time [25], leading to a significant burden on the respondents and healthcare team [26]. The 14-item Mediterranean Diet Score (MDS) was developed to assess adherence to the MDP [27], due to its effectiveness for primary and secondary prevention of CVDs [28-30]. In order to eliminate barriers for its use, a self-administered version of the MDS was developed and validated by Ghisi et al (2018) [31].

The prevalence and control of CVDs is considered the biggest public health challenge in Arabic-speaking countries [32], with risk factors such as obesity more than doubling in the last years [33]. Changes in food consumption patterns have been observed in these countries, including a diet with high content of fat, sugars, sodium and cholesterol [34,35]. In addition, availability of CR programs in Arab countries is extremely limited, with only 8 programs identified in a previous global survey of CR programs [36]. In this context, having a validated questionnaire to evaluate the MDP adherence in Arabic-speaking patients is timely, supporting practitioners to develop and deliver dietary recommendations that are aligned with patients’ needs. Therefore, this study aimed to translate and psychometrically validate the Arabic version of the MDS.

**Methods**

## **Design and Procedures**

The study was reviewed and approved by the King Abdullah Medical City, Saudi Arabia (22-944). Data was collected between September 2022 and January 2023. Permission to conduct this Arabic translation and validation was obtained, and the first author of the original MDS self-administered developmental study was invited to be part of the Arabic project. Authors had no access to information that could identify individual participants during or after data collection. Written informed consent was obtained from all participants for inclusion in the study.

Following best practices [37], this study included the following steps: 1) translation from English to Arabic and cultural adaptation of the MDS, 2) review by an expert panel and Arabic-speaking patients, and 3) cross-sectional survey for evaluation of psychometric properties.

As the first step, the initial conceptual translation of the scale from English to the target language (Arabic) was performed by an independent translator. Then, a second translator (now blinded to the objectives of the study) back translated the scale. All versions – original, translated, and back translated – were reviewed and combined by the research team into one version, which was reviewed by an expert team comprised of 10 healthcare providers that care for CVD patients (step 2). This expert team assessed face and content validity; the later by rating each item on a 5-point Likert-type scale ranging from 1 = completely irrelevant to 5 = very relevant. Content validity index (CVI) for the items (I-CVI) and scale (S-CVI) were computed. For both, only values higher than 0.80 were considered appropriate [38]. The scale was then revised based on CVI scores and suggestions made by experts. As part of phase 2, a group of 10 individuals from the target population assessed clarity of the scale usinf a 5-point Likert-type scale ranging from 1 = not clear to 5 = very clear. Results were analyzed to further revise the scale.

Finally, psychometric properties were evaluated (step 3). The translated and culturally adapted tool was administered to a convenience sample of cardiac patients from a cardiology center in Saudi Arabia. Patients were invited to participate in the study while attending regular clinical consultations. Those that accepted, signed the consent form, and completed the Arabic MDS in-person. The following psychometric properties were assessed following the Consensus-based Standards for the selection of health Measurement Instruments (COSMIN) taxonomy [39]: factor structure, internal consistency, construct, and criterion validity.

## **The self-administered version of the MDS**

The original 14-item MDS was developed in Spanish [27] and later translated to English [40]. The English version was used to develop the self-administered version [31]. This version included pictures to facilitate comprehension of questions and a simplified language. It is comprised of 13 items with yes/no options. The items are divided into 4 domains as follows: meats and processed foods; olive oil and sauce; fruits, vegetable, nuts, and legumes; and, fish and seafood [31]. Each item is scored as 0 or 1 in accordance with MDP adherence [41]. The final score ranges from 0 to 13 (< 5 = low adherence to MDP; > 10 = high adherence to MDP) [31]. The self-administered version of the MDS is also available in Brazilian [42] and Chinese [43] languages.

**Participants**

The inclusion criteria for patient participants were confirmed cardiac diagnosis or presence of cardiovascular risk factors, 18 years of age or older, and being able and willing to provide informed consent. The exclusion criteria for patient participants were inability to eat by mouth or having food and dietary restrictions (e.g., nut allergies) and having any impairment which preclude the participant’s ability to answer the questionnaire (e.g., visual). A minimum of 200 participants was required following the recommendation for factor analysis [39].

Patient participants self-reported their sociodemographic and clinical characteristics, including the following: age, sex, cardiovascular diagnosis and risk factors, living area, educational level, and family income. In addition, CR participation (yes/no) was self-reported to assess criterion validity.

**Data Analysis**

First, exploratory factor analysis was performed, using the main component method for factor extraction. Only factors with eigenvalues > 1.0 were considered. Item factor loadings > 0.3 were used in finalizing the items for each factor and interpreting the factors [44]. Next, internal consistency was determined by calculating Cronbach's alpha values of the scale and subscales. Cronbach's alpha higher than 0.70 was considered acceptable [45].

Criterion validity was assessed by differences in MDS total scores by CR participation using independent samples t-tests. Construct validity was assessed by exploring the associations between self-reported sociodemographic characteristics of participants and MDS scores using Pearson's correlation, independent samples t-tests, and analysis of variance.

Finally, a descriptive analysis of the Arabic MDS was performed. The Statistical Package for Social Sciences v. 28 (SPSS Inc., Chicago, IL, USA) was used for data analysis. The level of significance for all tests was set at 0.05.

**Results**

**Translation, cultural adaptation and review by an expert panel and patients**

Following translation and back translation, a revised Arabic MDS version was reviewed by an expert panel (n=10). The I-CVI ranged from 0.9 and 1.0 and the S-CVI was 0.9. These results identify an acceptable content validity for the Arabic MDS. For patients’ review (n=10), clarity scores ranged from 3.5 to 4.5/5 (mean 4.2±0.3). Given these results, two items were culturally adapted as follows: pork removed from items 10 and 11 due to religious and cultural reasons. In addition, items 6 and 13 were rephrased to increase clarity.

**Cross-sectional survey for evaluation of psychometric properties**

Overall, 200 patients completed the Arabic MDS. Their sociodemographic and clinical characteristics are presented in Table 1.

Results from the Kaiser-Meyer-Olkin index (KMO=0.78) and Bartlett’s Sphericity tests (*X²=*403.921, p<0.001) indicated that the data were suitable for exploratory factor analysis. Four factors were extracted, similar to the original validation [25]. These factors represented 62.3% of the total variance and were all internally consistent (Cronbach’s alpha ranged from 0.70-0.79). Factor 1 reflected items related to meats/processed foods (5 items), factor 2 related to olive oil/sauce items (3 items), factor 3 related fruits/vegetables and nuts/legumes (4 items), and factor 4 related to fish/seafood (1 item). Table 2 presents the factor structure of the Arabic MDS SV, including item loadings. The total Cronbach’s alpha of the scale was 0.74, confirming the internal consistency of the scale.

With regard to criterion validity, significant associations were observed between total scores and CR participation. Those who participated in CR had significantly higher adherence to the MDP (i.e., higher MDS scores) than those who did not participate (p=0.002; Table 1).

As also shown in Table 1, with regard to construct validity, there were significant associations between MDS scores and monthly family income (p=0.02), having the diagnosis of acute coronary syndrome (p=0.02), a history of valve repair or replacement (p=0.03), being obese (p=0.02) or having dyslipidemia (p=0.03). In this context, those with a family monthly income under SAR$10,000 presented a significantly lower MDP adherence than their counterparts. In addition, those who had a diagnosis of acute coronary syndrome, those that were obese, with dyslipidemia, or those who underwent a valve repair or replacement, presented MDP adherence than those without the diagnosis or a history of these procedures (Table 1).

**Adherence to the MDP**

The total score for the self-administered version of the MDS in this study was 11.0±8.0/13. Overall, 57 (28.5%) patients were classified as having high adherence to MDP and 29 (14.5%) as having low adherence. Table 2 also displays the number of patients that adhere to MDP recommendations (i.e., responded “yes” to each item asked in the questionnaire). Items with the highest adherence included seasoning foods with a combination of tomato, garlic, onions, or leek (n=171; 85.5%), eating poultry more often than red meat (n=164; 82.0%), and eating three servings or more of legumes a week (n=137; 68.5%). One item - eating three servings or more of fish or seafood each week – had the percentage of yes lower than 50% (n=87; 43.5%).

**Discussion**

Research has proven the benefits of the MDP for both primary and secondary prevention of CVDs [46]. However, multi-level barriers affect adherence to dietary recommendations, including in participants of CR programs [47]. Having a validated and convenient tool to assess MDP adherence – and ultimately guide clinical and educational practices – is timely in countries like Saudi Arabia where a small percentage of population meet dietary recommendations and programmes to improve dietary behaviours are urgently needed to reduce the current and future burden of disease [48]. Therefore, this study established the Arabic version of the MDS to assess adherence to the MDP through a multi-step process that included translation, cultural adaptation and assessment of psychometric properties. Content and face validity was supported based on experts and patients’ reviews. Subsequent factor analysis revealed 4 factors consistent with the original version of the instrument [31], all internally consistent. Criterion validity was confirmed by the significantly higher scores in patients who participated in CR. Construct validity was also established by significant associations between MDS scores and monthly family income, having the diagnosis of acute coronary syndrome or with a history of valve repair or replacement, being obese or having dyslipidemia. Overall, these results confirm the validity and reliability of the MDS in Arabic-speaking patients.

The self-administered version of the MDS – originally developed in English [31] and later translated and validated to Portuguese [41] and Chinese [42] – was developed to simplify the assessment of MDP adherence in clinical and research settings. This is the 4^th^ language available and other validation projects are underway (including Spanish and French). The Cronbach’s alpha of the Arabic MDS was 0.74, which is higher than previous studies (range: 0.42-0.69). The total MDS score of this score was higher than the Canadian (10.2±1.9) [31], Portuguese (6.94±1.99) [41], and Chinese (7.7±2.4) [42] versions. Items identified in the current study as the ones with the highest adherence included flavouring foods with a combination of tomato, garlic, onions, or leek, eating poultry more often than red meat, and eating three servings or more of legumes a week. These results are similar to responses identified in the previous studies using the MDS; in Canada [31], the items with highest adherence included eating poultry more often than meat and flavouring foods with a combination of tomato, garlic, onions or leek; in Brazil [41], eating three servings or more of legumes a week and flavoring foods with a combination of tomato, garlic, onions, or leeks; in China [42], flavouring foods with a combination of tomato, garlic, onions or leek. On the other hand, the self-reported consumption vegetable in the Arab-speaking sample was lower than the other samples (50% vs. 85% in Canada, 79% in Brazil and 87% in China) [31,41,42], despite the fact that the Saudi dietary guidelines strongly recommend a higher intake of vegetables [49].

As previously mentioned, construct validity of the Arabic MDS was confirmed in this study, as well as in other versions of this scale [31,41,42]. Specifically, those with lower monthly family income had significantly lower MDP adherence than their counterparts. Despite its complexity, the impact of socioeconomic on dietary pattern is undeniably important [50-52]. In general, people with higher income tend to follow the MDP recommendations [53]; however, in any context, recommendations to follow healthy dietary habits should be given to CVD patients. Future research examining the changes in adherence to the MD over time and after educational interventions is warrantied, including in groups of individuals with low socioeconomic status.

Those who participated in CR had significantly higher adherence to the MDP (i.e., higher MDS scores) than those who did not participate. These results support the effects of participation in CR – and delivery of nutrition counselling – on adherence to heart-healthy dietary patterns [54]. Furthermore, studies should describe the strategies, tools, and techniques employed within the interventions used to adhere to the MDP and barriers and facilitators to follow this behaviour.

There are some limitations in this study. First, findings are limited to one center from 1 Arabic-speaking country (Saudi Arabia); results cannot be generalizable to the other 21 countries in the world that speak Arabic. Second, convenience sampling was chosen due to limitations in time to conduct this study; therefore, there may be selection and retention bias, which also limits the generalizability of the findings. Third, because of the nature of this cross-sectional study, causal conclusions cannot be drawn; it is recommended to test if the scale is sensitive to change before and after a nutritional or educational intervention. The final limitation pertains to the assessment of other psychometric properties such as test-retest reliability, which should be explored in future studies.

**Conclusion**

In conclusion, the Arabic MDS is a valid and reliable tool that can be used to assess MDP adherence of Arabic-speaking cardiac patients. It is hoped that this scale supports the adequate assessment of food intake and changes in dietary habits, which plays a highly significant role in the care of people living with a cardiovascular condition.

**References**

1. Roth GA, Mensah, GA, Johnson, CO, et al. Global Burden of Cardiovascular Diseases and Risk Factors, 1990–2019. J Am Coll Cardiol. 2020; 76 (25): 2982–3021. <https://doi.org/10.1016/j.jacc.2020.11.010>.

2. Aljefree N, Ahmed F. Prevalence of Cardiovascular Disease and Associated Risk Factors among Adult Population in the Gulf Region: A Systematic Review. Adv Public Health. 2015; 2015:1–23. <https://doi.org/10.1155/2015/235101>.

3. Ahmed AM, Hersi A, Mashhoud W, et al. Cardiovascular Risk Factors Burden in Saudi Arabia: The Africa Middle East Cardiovascular Epidemiological (ACE) Study. J Saudi Heart Assoc; 2017;29 (4): 235–43. <https://doi.org/10.1016/j.jsha.2017.03.004>.

4. Carter HE, Schofield D, Shrestha R. Productivity Costs of Cardiovascular Disease Mortality across Disease Types and Socioeconomic Groups. Open Heart. 2019; 6 (1): e000939. <https://doi.org/10.1136/openhrt-2018-000939>.

5. Oldridge NB. Economic Burden of Physical Inactivity: Healthcare Costs Associated with Cardiovascular Disease. Eur J Cardiovasc Prev Rehabil. 2008; 15 (2): 130–9. <https://doi.org/10.1097/HJR.0b013e3282f19d42>.

6. Salvatore FP, Spada A, Fortunato F, et al. Identification of Health Expenditures Determinants: A Model to Manage the Economic Burden of Cardiovascular Disease. IJERPH. 2021; 18 (9): 4652. <https://doi.org/10.3390/ijerph18094652>.

7. Piepol MF, Hoes AW, Agewall S, et al. 2016 European Guidelines on Cardiovascular Disease Prevention in Clinical Practice: The Sixth Joint Task Force of the European Society of Cardiology and Other Societies on Cardiovascular Disease Prevention in Clinical Practice (Constituted by Representatives of 10 Societies and by Invited Experts) Developed with the Special Contribution of the European Association for Cardiovascular Prevention & Rehabilitation (EACPR). Eur Heart J. 2016; 37 (29): 2315–81. <https://doi.org/10.1093/eurheartj/ehw106>.

8. Thomas RJ, King M, Lui K, et al. AACVPR/ACCF/AHA 2010 Update: Performance Measures on Cardiac Rehabilitation for Referral to Cardiac Rehabilitation/Secondary Prevention Services: A Report of the American Association of Cardiovascular and Pulmonary Rehabilitation and the American College of Cardiology Foundation/American Heart Association Task Force on Performance Measures (Writing Committee to Develop Clinical Performance Measures for Cardiac Rehabilitation). Circulation. 2010; 122 (13): 1342–50. <https://doi.org/10.1161/CIR.0b013e3181f5185b>.

9. Grace SL, Turk-Adawi KI, Contractor A, et al. Cardiac Rehabilitation Delivery Model for Low-Resource Settings. Heart. 2016; 102 (18): 1449–55. <https://doi.org/10.1136/heartjnl-2015-309209>.

10. Smith SC, Benjamin EJ, Bonow RO, et al. AHA/ACCF Secondary Prevention and Risk Reduction Therapy for Patients With Coronary and Other Atherosclerotic Vascular Disease: 2011 Update: A Guideline From the American Heart Association and American College of Cardiology Foundation. Circulation. 2011; 124 (22): 2458–73. <https://doi.org/10.1161/CIR.0b013e318235eb4d>.

11. Yusuf S, Joseph P, Rangarajan S, et al. Modifiable Risk Factors, Cardiovascular Disease, and Mortality in 155 722 Individuals from 21 High-Income, Middle-Income, and Low-Income Countries (PURE): A Prospective Cohort Study. Lancet. 2020; 395 (10226): 795–808. <https://doi.org/10.1016/S0140-6736(19)32008-2>.

12. Yusuf S, Hawken S, Ôunpuu S, et al. Effect of Potentially Modifiable Risk Factors Associated with Myocardial Infarction in 52 Countries (the INTERHEART Study): Case-Control Study. Lancet. 2004; 364 (9438): 937–52. <https://doi.org/10.1016/S0140-6736(04)17018-9>

13. Mehra VM, Gaalema DE, Pakosh M, et al. Systematic Review of Cardiac Rehabilitation Guidelines: Quality and Scope. Eur J Prev Cardiol. 2020; 27 (9): 912–28. <https://doi.org/10.1177/2047487319878958>.

14. Taylor RS, Dalal HM, McDonagh STJ. The Role of Cardiac Rehabilitation in Improving Cardiovascular Outcomes. Nat Rev Cardiol. 2022; 19 (3): 180–94. <https://doi.org/10.1038/s41569-021-00611-7>

15. Kabboul N, Tomlinson G, Francis T, et al. Comparative Effectiveness of the Core Components of Cardiac Rehabilitation on Mortality and Morbidity: A Systematic Review and Network Meta-Analysis. JCM. 2018; 7 (12): 514. <https://doi.org/10.3390/jcm7120514>

16. Shi W, Ghisi GLM, Zhang L, et al. Systematic Review, Meta‐analysis and Meta‐regression to Determine the Effects of Patient Education on Health Behaviour Change in Adults Diagnosed with Coronary Heart Disease. J Clin Nurs. 2022; jocn.16519. <https://doi.org/10.1111/jocn.16519>

17. Artinian NT, Fletcher GF, Mozaffarian D, et al. Interventions to Promote Physical Activity and Dietary Lifestyle Changes for Cardiovascular Risk Factor Reduction in Adults: A Scientific Statement From the American Heart Association. Circulation. 2010; 122 (4): 406–41. <https://doi.org/10.1161/CIR.0b013e3181e8edf1>

18. Ravera A, Carubelli V, Sciatti E, et al. Nutrition and Cardiovascular Disease: Finding the Perfect Recipe for Cardiovascular Health. Nutrients. 2016; 8(6): 363. <https://doi.org/10.3390/nu8060363>

19. Lichtenstein AH, Appel LJ, Vadiveloo M, et al. 2021 Dietary Guidance to Improve Cardiovascular Health: A Scientific Statement From the American Heart Association. Circulation. 2021; 144(23): e472–87. <https://doi.org/10.1161/CIR.0000000000001031>

20. Galbete C, Schwingshackl L, Schwedhelm C, et al. Evaluating Mediterranean diet and risk of chronic disease in cohort studies: an umbrella review of meta-analyses. Eur J Epidemiol. 2018; 33(10): 909–31. <https://doi.org/10.1007/s10654-018-0427-3>

21. Dinu M, Pagliai G, Casini A, et al. Mediterranean diet and multiple health outcomes: an umbrella review of meta-analyses of observational studies and randomised trials. Eur J Clin Nutr. 2018; 72(1): 30–43. <https://doi.org/10.1038/ejcn.2017.58>

22. Linan Pinto M, Pinto R, Charneca S, et al. Body composition, lipid profile and mediterranean diet adherence in cardiovascular disease patients attending a long-term exercise-based cardiac rehabilitation program during COVID-19 pandemic. Eur J Prev Cardiol. 2021; 28(Suppl 1): zwab061.187. <https://doi.org/10.1093/eurjpc/zwab061.187>

23. Della Valle PG, Mosconi G, Nucci D, et al. Adherence to the Mediterranean Diet during the COVID-19 national lockdowns: a systematic review of observational studies. Acta bio-medica Atenei Parmensis. 2021; 92(S6): e2021440. <https://doi.org/10.23750/abm.v92iS6.12233>

24. Testa MA, Simonson DC. Chapter 12 - The Use of Questionnaires and Surveys. In Clinical and Translational Science (Second Edition); Robertson D, Williams GH. Eds.; Academic Press, 2017; 207–26. <https://doi.org/10.1016/B978-0-12-802101-9.00012-0>

25. Willett W. Future Directions in the Development of Food-Frequency Questionnaires. Am J Clin Nutr. 1994; 59 (1): S171–4. <https://doi.org/10.1093/ajcn/59.1.171S>

26. Kowalkowska J, Slowinska M, Slowinski D, et al. Comparison of a Full Food-Frequency Questionnaire with the Three-Day Unweighted Food Records in Young Polish Adult Women: Implications for Dietary Assessment. Nutrients. 2013; 5 (7): 2747–76. <https://doi.org/10.3390/nu5072747>

27. Martínez-González MA, Salas-Salvadó J, Estruch R, et al. Benefits of the Mediterranean Diet: Insights From the PREDIMED Study. Prog Cardiovasc Dis. 2015; 58 (1): 50–60. <https://doi.org/10.1016/j.pcad.2015.04.003>

28. Martinez-Gonzalez MA, Bes-Rastrollo M. Dietary Patterns, Mediterranean Diet, and Cardiovascular Disease. Curr Opin Lipidol. 2014; 25 (1): 20–6. <https://doi.org/10.1097/MOL.0000000000000044>

29. Jimenez-Torres J, Alcalá-Diaz JF, Torres-Peña JD, et al. Mediterranean Diet Reduces Atherosclerosis Progression in Coronary Heart Disease: An Analysis of the CORDIOPREV Randomized Controlled Trial. Stroke. 2021; 52 (11): 3440–9. <https://doi.org/10.1161/STROKEAHA.120.033214>

30. Guasch‐Ferré M, Willett WC. The Mediterranean Diet and Health: A Comprehensive Overview. J Intern Med. 2021; 290 (3): 549–66. <https://doi.org/10.1111/joim.13333>

31. Ghisi GLDM, Mahajan A, Da Silva Chaves GS, et al. Validation of a Self-Administered Version of the Mediterranean Diet Scale (MDS) for Cardiac Rehabilitation Patients in Canada. Int J Food Sci Nutr. 2019; 70 (2): 202–11. <https://doi.org/10.1080/09637486.2018.1486392>

32. Elmusharaf K, Grafton D, Jung JS, et al. The Case for Investing in the Prevention and Control of Non-Communicable Diseases in the Six Countries of the Gulf Cooperation Council: An Economic Evaluation. BMJ Global Health. 2022; 7 (6): e008670. <https://doi.org/10.1136/bmjgh-2022-008670>

33. World Heart Organization. WHO Global Health Observatory: Prevalence of obesity among adults, BMI ≥ 30, age-standardized estimates by country. [internet]. 2016. Accessed 3 May 2023. Available from: <https://www.who.int/data/gho/data/themes/topics/indicator-groups/indicator-group-details/GHO/bmi-among-adults>

34. Musaiger AO. Diet and Prevention of Coronary Heart Disease in the Arab Middle East Countries. Med Princ Pract. 2002; 11 (Suppl. 2): 9–16. <https://doi.org/10.1159/000066415>

35. Al Moraie N, Lietz G, Seal CJ. Dietary Patterns and Risk of Heart Disease in Populations from Different Geographical Locations in Saudi Arabia. Proc Nutr Soc. 2012; 71 (OCE2): E50. <https://doi.org/10.1017/S0029665112001073>

36. Turk-Adawi KI, Terzic C, Bjarnason-Wehrens B, et al. Cardiac Rehabilitation in Canada and Arab Countries: Comparing Availability and Program Characteristics. BMC Health Serv Res. 2015; 15 (1): 521. <https://doi.org/10.1186/s12913-015-1183-7>

37. Wild D, Grove A, Martin M, et al. Principles of Good Practice for the Translation and Cultural Adaptation Process for Patient-Reported Outcomes (PRO) Measures: Report of the ISPOR Task Force for Translation and Cultural Adaptation. Value Health. 2005; 8 (2): 94–104. <https://doi.org/10.1111/j.1524-4733.2005.04054.x>

38. Mastaglia B, Toye C, Kristjanson LJ. Ensuring Content Validity in Instrument Development: Challenges and Innovative Approaches. Contemporary Nurse. 2003; 14 (3): 281–91. <https://doi.org/10.5172/conu.14.3.281>

39. Terwee CB, Bot SDM, De Boer MR, et al. Quality Criteria Were Proposed for Measurement Properties of Health Status Questionnaires. J Clin Epidemiol. 2007; 60 (1): 34–42. <https://doi.org/10.1016/j.jclinepi.2006.03.012>

40. BACPR. The BACPR Standards and Core Components for Cardiovascular Disease Prevention and Rehabilitation [internet]. 2023. Accessed 3 May 2023. Available from: <https://www.bacpr.org/__data/assets/pdf_file/0021/64236/BACPR-Standards-and-Core-Components-2023.pdf>

41. Schröder H, Fitó M, Estruch R, et al. A Short Screener Is Valid for Assessing Mediterranean Diet Adherence among Older Spanish Men and Women. Journal Nutr. 2011; 141 (6): 1140–5. <https://doi.org/10.3945/jn.110.135566>

42. Teixeira R, Jansen AK, Pereira DA, et al. Brazilian Portuguese Version of the Mediterranean Diet Scale: Translation Procedures and Measurement Properties. Diabetes Metabol Syndr: Clin Res Rev. 2021; 15 (4): 102165. <https://doi.org/10.1016/j.dsx.2021.06.002>

43. Li J, Ding H, Wang Z, et al. Translation, Cultural Adaptation, Reliability, and Validity Testing of a Chinese Version of the Self-Administered Mediterranean Diet Scale. Front Nutr. 2022; 9: 831109. <https://doi.org/10.3389/fnut.2022.831109>

44. Hair JF, Anderson RE. Multivariate data analysis. New Jersey: Prentice Hall, 1998.

45. Nunnally JC. Pyschometric Theory. McGraw-Hill: New York, United States, 1978.

46. Widmer RJ, Flammer AJ, Lerman LO, et al. The Mediterranean Diet, Its Components, and Cardiovascular Disease. Am J Med. 2015; 128 (3): 229–38. <https://doi.org/10.1016/j.amjmed.2014.10.014>

47. Vanzella LM, Rouse V, Ajwani F, et al. Barriers and Facilitators to Participant Adherence of Dietary Recommendations within Comprehensive Cardiac Rehabilitation Programmes: A Systematic Review. Public Health Nutr. 2021; 24 (15): 4823–39. <https://doi.org/10.1017/S1368980021002962>

48. Moradi-Lakeh M, El Bcheraoui C, Afshin A, et al. Diet in Saudi Arabia: Findings from a Nationally Representative Survey. Public Health Nutr. 2017; 20 (6): 1075–81. <https://doi.org/10.1017/S1368980016003141>

49. Ministry of Health. The Dietary Guidelines for Saudis. [internet] 2023. Accessed 3 May 2023. Available from: <https://www.moh.gov.sa/en/HealthAwareness/Pages/SaudihealthFoodGuide.aspx>

50. Mayén AL, Marques-Vidal P, Paccaud F, et al. Socioeconomic Determinants of Dietary Patterns in Low- and Middle-Income Countries: A Systematic Review. Am J Clin Nutr. 2014; 100 (6): 1520–31. <https://doi.org/10.3945/ajcn.114.089029>

51. D’Innocenzo S, Biagi C, Lanari M. Obesity and the Mediterranean Diet: A Review of Evidence of the Role and Sustainability of the Mediterranean Diet. Nutrients. 2019; 11 (6): 1306. <https://doi.org/10.3390/nu11061306>

52. Vlismas K, Stavrinos V, Panagiotakos DB. Socio-Economic Status, Dietary Habits and Health-Related Outcomes in Various Parts of the World: A Review. Cent Eur J Public Health. 2009; 17 (2): 55–63. <https://doi.org/10.21101/cejph.a3475>

53. Cavaliere A, Elisa De Marchi A, Banterle A. Exploring the Adherence to the Mediterranean Diet and Its Relationship with Individual Lifestyle: The Role of Healthy Behaviors, Pro-Environmental Behaviors, Income, and Education. Nutrients. 2018; 10 (2): 141. <https://doi.org/10.3390/nu10020141>

54. Kocanda L, Schumacher TL, Plotnikoff RC, et al. Effectiveness and reporting of nutrition interventions in cardiac rehabilitation programmes: a systematic review. Eur J Cardiovasc Nurs. 2023; 22(1): 1–12. <https://doi.org/10.1093/eurjcn/zvac033>

**Tables**

**Table 1: Characteristics of patient participants and corresponding mean total MDS scores (N=200)**

| **Characteristic** |  | **MDS Total Score** mean±SD | **p*** |
| --- | --- | --- | --- |
| *Age,* mean±SD | 46.5±13.6 | - | - |
| *Sex,* n (%) |  |  | 0.11 |
| Male | 92 (46.0) | 8.3±2.3 |  |
| Female | 108 (54.0) | 7.7±2.4 |  |
| *Area of Living,* n (%) |  |  | 0.51 |
| Urban | 180 (90.0) | 7.9±2.4 |  |
| Rural | 19 (9.5) | 8.5±1.7 |  |
| *Highest educational level,* n (%) |  |  | 0.91 |
| Illiterate | 9 (4.5) | 7.7±2.7 |  |
| Less than High School | 5 (2.5) | 8.2±1.9 |  |
| High School | 62 (31.0) | 7.8±2.5 |  |
| College certificate or diploma | 109 (54.5) | 8.0±2.3 |  |
| Higher education | 15 (7.5) | 8.3±2.0 |  |
| *Monthly family income,* n (%) |  |  | 0.02 |
| Under SAR$10,000 | 78 (39.0) | 6.6±2.5 |  |
| Between SAR$10,001-20,000 | 76 (38.0) | 7.6±2.2 |  |
| Between SAR$20,001-$30,000 | 38 (19.0) | 7.5±2.4 |  |
| Higher than SAR$30,001 | 8 (4.0) | 9.1±2.2 |  |
| *Cardiac Diagnosis/Procedures,* n (%) |  |  |  |
| Percutaneous Coronary Intervention | 24 (12.0) | 8.1±2.2 | 0.72 |
| Arrhythmia | 22 (11.0) | 7.4±2.6 | 0.25 |
| Acute Coronary Syndrome | 21 (10.5) | 9.0±2.2 | 0.02 |
| Acute Myocardial Infarction | 16 (8.0) | 8.0±2.3 | 0.90 |
| Valve disorders | 16 (8.0) | 8.5±3.3 | 0.40 |
| Cardiac Device | 11 (5.5) | 8.1±2.2 | 0.85 |
| Valve Repair/Replacement | 9 (4.5) | 9.4±3.2 | 0.03 |
| Heart Failure | 8 (4.0) | 7.4±1.6 | 0.50 |
| Coronary Artery Bypass Graft | 2 (1.0) | 7.5±2.1 | 0.78 |
| *Risk factors,* n (%) |  |  |  |
| Family History of CVD | 79 (39.5) | 7.7±2.4 | 0.30 |
| Sedentary/inactivity | 72 (36.0) | 7.8±2.5 | 0.50 |
| Smoking | 64 (32.0) | 8.1±2.1 | 0.70 |
| Diabetes Type II | 60 (30.0) | 8.3±2.3 | 0.23 |
| Hypertension | 46 (23.0) | 7.8±2.4 | 0.52 |
| Obesity | 44 (22.0) | 5.7±2.3 | 0.02 |
| Depression | 43 (21.5) | 8.2±2.5 | 0.44 |
| Dyslipidemia | 34 (17.0) | 6.5±2.3 | 0.03 |
| *CR participation,* yes (%) |  |  | 0.002 |
| Yes | 22 (11.0) | 9.5±2.2 |  |
| No | 178 (89.0) | 7.6±2.3 |  |

MDS, Mediterranean Diet Scale; SD standard deviation; CVD cardiovascular diseases; CR cardiac rehabilitation.

*p is used for association of characteristics and total mean score, tested using t-tests, analysis of variance or Pearson’s correlation, as applicable.

Note: Income shown in Saudi Riyal (SAR). 1 SAR corresponds to USD$0.47 (currency: March 1, 2023)

**Table 2: Factor loadings from exploratory factor analysis, reliability of factors and number of ‘yes’ responses per item, N=202**

| **Item** | **Factors** | | | | **Yes, n (%)** |
| --- | --- | --- | --- | --- | --- |
|  | **1. Meats and Processed Foods** | **2. Olive Oil and Sauces** | **3. Fruits, Vegetables, Legumes and Nuts** | **4. Fish and Seafood** |  |
| 1. Do you use olive oil as the main source of fat when you cook? |  | 0.721 |  |  | 123 (61.5) |
| 2. Do you use at least four tablespoons or more of olive oil when cooking your food each day? |  | 0.733 |  |  | 118 (59.0) |
| 3. Do you eat two servings or more of vegetables each day? |  |  | 0.689 |  | 100 (50.0) |
| 4. Do you eat three servings or more of fruit each day? |  |  | 0.629 |  | 101 (50.5) |
| 5. Do you eat less than one tablespoon of butter, hydrogenated margarine or cream each day? | 0.466 |  |  |  | 111 (55.5) |
| 6. Do you drink less than one serving of sweet or sweetened drinks each day? | 0.408 |  |  |  | 100 (50.0) |
| 7. Do you eat three servings or more of legumes a week? |  |  | 0.469 |  | 137 (68.5) |
| 8. Do you eat three servings or more of fish or seafood each week? |  |  |  | 0.474 | 87 (43.5) |
| 9. Do you eat one serving or more of nuts each week? |  |  | 0.453 |  | 129 (64.5) |
| 10. Do you eat poultry (chicken or turkey) more often than meat (beef, veal, hamburger, or sausage)?* | 0.453 |  |  |  | 164 (82.0) |
| 11. Do you limit red meat and processed meats to one serving or less one or two times a week?* | 0.452 |  |  |  | 132 (66.0) |
| 12. Do you eat less than three servings of sweets or pastries each week? | 0.405 |  |  |  | 119 (59.5) |
| 13. Do you add some flavorings to your food, such as a mixture of tomatoes, garlic, onions and leeks, two or more times a week? |  | 0.652 |  |  | 171 (85.5) |
| Variance explained | 27.4% | 16.8% | 9.6% | 8.5% | - |
| Eigenvalues | 2.9 | 1.5 | 1.3 | 1.1 | - |
| Reliability | 0.79 | 0.78 | 0.75 | 0.70 | - |

* Item culturally adapted.
